# Exercise Intensity Influences Prefrontal Cortex Oxygenation during Cognitive Testing

**DOI:** 10.3390/bs9080083

**Published:** 2019-07-26

**Authors:** Terence Moriarty, Kelsey Bourbeau, Bryanne Bellovary, Micah N. Zuhl

**Affiliations:** 1 Department of Health, Exercise, and Sports Sciences, University of New Mexico, Albuquerque, NM 87131, USA; 2 Department of Kinesiology, University of Northern Iowa, Cedar Falls, IA 50614, USA; 3 School of Health Sciences, Central Michigan University, Mount Pleasant, MI 48859, USA

**Keywords:** aerobic exercise, yoga, prefrontal cortex, oxygenation, cognition

## Abstract

Activation changes in the prefrontal cortex (PFC) regions have been linked to acute exercise-induced improvements in cognitive performance. The type of exercise performed may influence PFC activation, and further impact cognitive function. The present study aimed to compare PFC activation during cognitive testing after moderate-intensity, high intensity, and yoga exercises, and to determine if PFC activation is linked to cognitive performance. Eight subjects (four male and four female), aged 35 ± 5 completed a control, high intensity, moderate intensity, and yoga exercises followed by administration of a cognitive task (NIH Toolbox Fluid Cognition). Left and right PFC activation (LPFC and RPFC, respectively) were evaluated by measuring hemoglobin difference (Hbdiff) changes during post-exercise cognitive assessment using functional near infrared spectroscopy (fNIRS). Activation during the cognitive test was higher in the LPFC after moderate intensity exercise compared to control, high intensity, and yoga (5.30 ± 6.65 vs. 2.26 ± 2.40, 2.50 ± 1.48, 2.41 ± 2.36 μM, *p* < 0.05, respectively). A negative relationship was detected between LPFC and processing speed after exercise. PFC activation did not align with cognitive performance. However, acute exercise, regardless of type, appeared to alter neural processing. Specifically, less PFC activation was required for a given neural output after exercise.

## 1. Introduction

A single bout of aerobic exercise has been shown to influence neurophysiological pathways that promote heightened post-exercise cognitive functioning such as processing speed, working memory, and executive function [1]. Low to moderate intensity, along with high intensity bouts of exercise have reportedly improved performance in various cognitive constructs [2]. Further, single sessions of mind-body therapies such as yoga have led to memory and processing speed improvements [3]. One mechanism that possibly explains acute exercise effects on cognitive function is through activation changes in the prefrontal cortex (PFC) of the brain [4]. The dorsal lateral (left and right) prefrontal cortex (DLPFC) is thought to be responsible for cognitive control and goal-directed behavior. The DLPFC is highly active during memory retrieval and in response to mentally arduous tasks [5,6]. The left-DLPFC has been associated with processing speed and executive function, and appears to be influenced by acute exercise [7,8]. 

Heightened post-exercise cognitive functioning has been linked to greater activation in the DLPFC, which indicates increased concentration or mental focus after exercise [8,9]. For example, a single thirty second bout of high intensity cycling until exhaustion increased DLPFC activity during post-exercise cognitive testing among healthy adults [2]. Similarly, ten minutes of moderate intensity exercise at 50% maximal oxygen consumption (VO_2_max) was effective in activating the left-DLPFC which aligned with improved reaction time performance among older individuals [10]. Conversely, lower post-exercise cortical activation has been associated with an increase or no change in cognitive performance [11]. Reduced PFC activity in the face of improved, or non-changing cognitive function, is considered to be a neural efficiency adaptation to a stimulus [12]. For example, healthy adults demonstrated lower DLFC oxygenation along with improvements in processing speed after each session of a transcranial stimulation exercise [13]. Within this framework, lesser mental effort is needed for mental output performance [14]. Likewise, higher cortical activity occurring along with a decrease in cognitive function may indicate reduced neural efficiency, and highlights greater neural input required for a given cognitive task [12]. 

Considerable efforts have been made to investigate the underlying mechanisms of PFC activation during exercise that lead to improved brain function for several minutes after the exercise bout [15]. During low to moderate intensity exercise, there is an increase in cerebral blood flow and oxygenation, which may promote distribution of nutrients throughout the brain, and also induce arousal during subsequent cognitive testing [16]. When exercise reaches higher intensities, resources are shifted towards brain areas of motor function, and away from regions of cognitive processing, which includes the PFC [17]. This response has been termed transient hypofrontality, and may lead to downstream cognitive improvements due to reperfusion of cortical areas during later mental tasks [17]. As previously stated, both high and low intensity aerobic exercise bouts have induced PFC activation and improved post-exercise cognitive functioning [8,9]. Yoga, which incorporates muscle stretching and breathing exercises is considered a low intensity form of mind-body exercise, has also improved PFC activation and cognition [18]. While comparisons of PFC activation during various types of exercises has been performed [19,20], less research efforts have been made into contrasting PFC activation during cognitive testing after different types of exercise. It is important to examine and compare the types of acute exercise (high or moderate intensity and yoga) that influence post-exercise PFC activation changes during concentration tasks, and also evaluate if the PFC activation aligns with post-exercise cognitive performance (e.g., processing speed and episodic memory). Understanding this connection between PFC activity and cognition would provide insight into neural efficiency changes in response to exercise. 

The post-exercise measurement of neural activation during a cognitive task may help to explain how exercise rapidly modulates cognition. Functional near-infrared spectroscopy (fNIRS) is a noninvasive method designed to monitor brain blood flow by quantifying changes in oxygenated and deoxygenated hemoglobin in the brain [21]. The fNIRS measurement is able to discriminate between varying mental efforts, and may provide an indirect marker of neural activation [22]. A cap with various probe configurations is placed on the head of a subject to record activity in the brain. The configuration of the cap allows researchers to examine brain oxygenation changes in targeted cortical regions. The fNIRS technology has been used to measure post-exercise changes in oxygenation, or neural activation within the DLPFC while a participant performs an attention demanding task [2,4,20,23,24]. The use of fNIRS technology in the current study allows us to determine which type of exercise alters neural oxygenation changes (i.e., activation) of the PFC during post-exercise cognitive testing. 

Another possible cause of exercise-induced rapid enhancement in cognitive performance is through upregulation of neurotrophins such as brain derived neurotrophic factor (BDNF), which is a neural growth factor attributed to promoting memory enhancing benefits [25,26,27]. Limited results have suggested that higher intensity exercise leads to BDNF expression [2,28]. A link between the exercise metabolite, lactate, which when elevated indicates greater metabolic stress, and plasma BDNF has also been shown [28]. Interestingly, infusing lactate into a resting human induced BDNF release from skeletal muscle [29]. Currently, limited research has been performed in which brain imaging (fNIRS), along with cognitive, neurogenesis (BDNF), metabolic (lactate), and cardiovascular (HR) measures were compared between various types of exercise. Therefore, the primary aim of the study is to evaluate PFC oxygenation changes during cognitive tasks performed after acute bouts of moderate intensity, high intensity, and yoga exercises, and to further determine the relationship between post-exercise PFC oxygenation and cognitive performance. This will provide insight into neural efficiency changes in response to exercise. We also aim to determine if markers of cardiovascular stress and metabolic stress during exercise, and BDNF expression post-exercise are related to cognitive changes after each exercise session. 

## 2. Materials and Methods

Subjects: All study procedures are in accordance with the Code of Ethics of the World Medical Association for experiments using human participants. Participants were recruited from university staff and via flyers posted at urban sports complexes. Eight total (men = 4, women = 4) participants volunteered to take part in this study (Table 1). All completed a health questionnaire, and procedures, discomforts, and risks were discussed before written informed consent was obtained. Physically active (three or more days per week of aerobic exercise, but no yoga experience) men and women between the ages of 18–44 were recruited. Overall, participants completed 1–2 h per week of various activities, including: team sport practice and competition, aerobic exercise, and strength training. The participants reported no cardiovascular, pulmonary or metabolic disorders. Subjects had no known behavioral, psychological or neural disorders, and were also excluded if they were taking antidepressant medications. All study procedures were performed in the Exercise Laboratory at the University of New Mexico (UNM) and the protocol (03318) was approved by the UNM Institutional Review Board for Human Subject Research.

Study Protocol: Each participant completed baseline testing, followed by four trials completed in randomized order. The four conditions included, non-exercise control (C), moderate intensity aerobic exercise (MIE), high intensity aerobic interval exercise (HIE), and mind-body yoga exercise (YE). Baseline and all exercise trials were separated by more than 72 h. Participants arrived for baseline and exercise 2 h post-prandial. Baseline measurements included maximal oxygen consumption (VO_2_max), body composition via skinfolds, and 15 mL blood sample. Upon arrival for the exercise trials, subjects were seated and fitted with the fNIRS cap to collect 10 min pre-exercise data. The purpose for pre-exercise fNIRS measure was to determine if oxygenation changes during cognitive testing increased after exercise within each trial. Then a heart rate strap and watch were donned (Polar, Oulu, Finland), and a measure of resting whole blood lactate and oxygen consumption (VO_2_) was completed. All exercise conditions (MIE, HIE, and YE) were 45 min in duration with measurements of heart rate, VO_2_, lactate, and RPE performed throughout each trial. After completion of exercise, and upon heart rate returning to resting values, but no sooner than twenty minutes post-exercise, the participant was fitted with a head cap containing optodes for fNIRS monitoring of the right and left prefrontal cortex regions. Once fitted with the fNIRS, the NIH Toolbox fluid cognition battery assessment was administered. Lastly, a 15 mL blood sample was collected after completion of the cognitive assessment.

### 2.1. Baseline Measures

VO_2_max (Maximal Oxygen Consumption Test): All tests were performed on an electronically-braked cycle ergometer (Lode, Groningen, The Netherlands). After a brief warm-up, subjects were connected to a metabolic cart (True One; Parvomedics, Sandy, UT, USA) for the continuous measurement of oxygen consumption and carbon dioxide production. During each test, workload was increased every second until subjects reached volitional fatigue, or cadence dropped below 60 rpm. The goal of the test was for subjects to reach maximal effort within 8–10 min. VO_2_max required two of the following four criteria to be met: respiratory exchange ratio (RER) > 1.15, within ±10 bpm of age-predicted maximal HR, VO_2_ plateau of ≤150 mL·min^−1^, or RPE > 17. Maximal oxygen consumption was determined based on the highest value achieved using an 11-breath rolling average.

Body Composition: A three-site skinfold measurement was performed to estimate body density (Lange; Beta Technology Inc., Cambridge, MD, USA) and used to determine percent body fat using the appropriate Jackson equation for men and women [30]. Each site was measured twice and if not within 2 mm, a third measurement was done. The average of the two closest values were used for the calculation. Height and weight were measured using a stadiometer and floor scale, respectively, and further used to calculate body mass index (BMI).

### 2.2. Exercise Protocols

Moderate Intensity Aerobic Exercise (MIE): The protocol was completed using an electronically braked cycle ergometer (Lode, Groningen, The Netherlands). Resting measurements of whole blood lactate, heart rate, and VO_2_ were performed, and then 5-min warm-up was completed at a self-selected workload. At the end of warm-up, heart rate, VO_2_ (recorded from 1-min sample of expired gases), RPE, and lactate were measured. Participants then cycled for 30 continuous minutes at 50% intensity as identified by the workload (in watts) achieved at 50% of VO_2_max. Heart rate and RPE were recorded at every 5-min interval. The participants were encouraged to achieve a moderate intensity of exercise, by using the Borg scale (6–20), which measures perceived exertion. A rating of 12 on this scale, was recommended as equivalent to the exertion in moderate intensity aerobic exercise [31]. Workload was adjusted if RPE exceeded 13 to maintain the desired intensity of exercise. Oxygen consumption (VO_2_) was recorded every 10 min, and lactate was measured at the end of the 30-min cycling bout. A 10-min cool down was completed at a self-selected workload for total exercise time of 45 min. The fNIRS measurement and NIH Toolbox cognitive assessment were performed when heart rate returned to pre-exercise levels, but not before 20 min of rest. The 20-min rest period prior to cognitive testing was chosen based on previous meta-analytical results which reported a large positive effect of exercise on cognitive function following 20 min of exercise [1]. A 15 mL blood sample was collected after completion of the cognitive assessment. 

High Intensity Aerobic Interval Exercise (HIE): All procedures through the end of the 5-min warm-up phase were identical to MIE. Participants completed 1-min cycling bouts at 85–95% of workload achieved during VO_2_max test followed by 2 min of recovery at 40% intensity. This work to rest ratio (1:2) was performed for 30 min and included 10 intervals. Heart rate and RPE were recorded at the end of each interval, and workload was adjusted if RPE exceeded 17 (6–20 scale). Lactate was measured at the end of the final interval. A 1-min VO_2_ sample was recorded every 10 min. A 10-min cool down was completed at a self-selected workload (total exercise time was 45 min). The fNIRS measurement combined with the NIH Toolbox cognition test was completed when heart rate returned to pre-exercise values, but not before 20 min. A 15 mL blood sample collection was collected upon completion of the cognitive assessment.

Mind-Body Yoga Exercise (YE): The yoga exercise was 45 min in duration (matched to MIE and HIE) and standardized through both a video and audio recording. Heart rate, RPE, VO_2_, and lactate were recorded at the same time points as the MIE and HIE trials. The yoga consisted of 21 specific bodily postures held for varying time combined with breathing and meditation. Specific poses included: 1. Sun salutations; 2. Eagle; 3. Sanding head to knee; 4. Balancing stick; 5. Standing bow; 6. Standing separate leg; 7. Triangle; 8. Standing separate leg-head to knee; 9. Cobra; 10. Locust both legs; 11. Downward dog leg extension; 12. Plank; 13. Knee to nose; 14. Hip stacking; 15. Hip stacking; 16. Child’s; 17. Camel; 18. Butterfly; 19. Wind removing both legs; 20. Happy baby; and 21. Corpse. Twenty minutes post yoga and when heart returned to resting value, the fNIRS measurement combined with the NIH Toolbox cognition test was performed. A 15 mL blood sample collection was collected upon completion of the cognitive assessment.

Non-Exercise Control (C): The control period matched the duration of the MIE, HIE, and YE conditions. Upon participant arrival, they were seated for 65 min (matched exercise + 20-min post-exercise recovery). During the control period they were instructed to sit quietly without access to technological devices (e.g., computers, smart phones), and had limited social engagement. Afterwards, the fNIRS measurement combined with the NIH Toolbox cognition test was performed. A 15 mL blood sample collection was collected upon completion of the cognitive assessment. 

**Figure 1:**
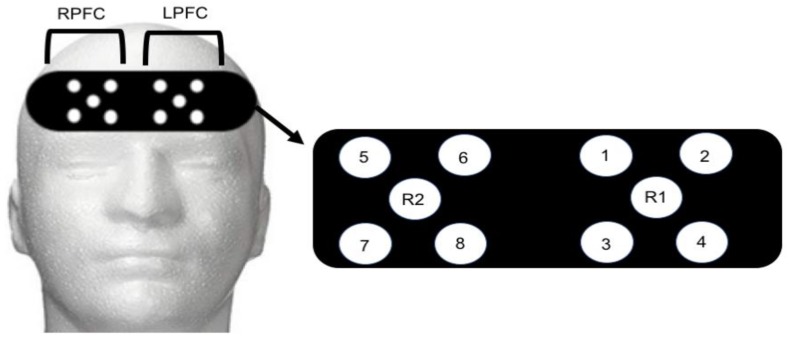
Schematic representation of functional near-infrared spectroscopy optodes. Open circles with numbers (1–8) represent light sources, and open circles with R1 and R2 represent receivers. Overall, 8 channels were created with 1–4 monitoring the LPFC and 5–8 monitoring the RPFC.

Functional Near Infrared Spectroscopy Recording: An 8-channel continuous wave fNIRS system (OctaMon, Artinis Medical Systems, Elst, The Netherlands) was used to capture brain activity during control and post-exercise administration of the NIH Toolbox Fluid Cognition assessment. Four LED optodes combined with one receiver were placed over the right hemisphere (4 transmitters, 1 receiver) and left hemisphere (4 transmitters, 1 receiver) of the prefrontal cortex (8 × 2 configuration). Optode placement was based on the modified international electroencephalogram 10–20 system [32,33]. Regions of LPFC included Fp1 and F7 with the readings from channels 1–4 (Figure 1). Regions of RPFC included Fp2 and F7 with readings from channels 5–8 (Figure 1). The measurement locations were identified by locating the nasion site and placing the edge of the cap 2 cm above this point (roughly 1 cm above brow line) and centering on the measured Fpz location [34]. Inter-optode distance was 3.5 cm and signal sampling of 10 Hz was used. Transmitting wavelengths were ±760 nm and ±850 nm. The LPFC site most likely contained both the left-DLPFC and left-frontal lateral PFC. Similarly, the RPFC contained the right-DLPFC and right-frontal lateral PFC. The fNIRS signal was band-pass filtered at 0.01 to 0.50 Hz to remove physiological artefact [33,35]. Relative concentration changes for oxyhemoglobin (HbO_2_) and deoxyhemoglobin (HHb) were measured from resting baseline within each trial using the first 10 s, and defined as 0 µmol. A 10-min control period was collected before each exercise trial to monitor pre-exercise values. The fNIRs device is capable of quantifying frontal cortex oxygenation and blood flow changes [36]. The most sensitive measure of cerebral oxygenation is the oxyhemoglobin difference (Hbdiff) due to the high correlation with cerebral blood flow and mean arterial pressure changes [36,37]. Hbdiff has a linear relationship with both mean arterial pressure, cerebral blood flow, and flow velocities; and, has been used as a marker of cerebral hemorrhage, and hypotension in critically ill patients [37]. Therefore, the Hbdiff was used to evaluate brain blood flow and thus oxygenation during the cognitive task [38].

Cognitive Task: Cognition was evaluated during control and post-exercise conditions using the NIH Toolbox Assessment of Neurological and Behavioral Function within the cognitive domain [39]. The fluid cognition battery for ages 3–85 years was delivered using an iPad device, and consisted of five measurements, including: 1. Flanker inhibitory control and attention test measuring executive function and attention constructs; 2. Dimensional change card sort tests measuring executive function construct; 3. Picture sequence memory test measuring episodic memory construct; 4. List sorting work memory test measuring working memory cognition construct; and 5. Pattern comparison processing speed test measuring processing speed construct. The fluid cognition battery was chosen because it is considered a more global assessment of abilities to solve problems, think, and develop episodic memories. Fluid abilities appear to be more responsive to changes in brain functioning as a result of an intervention (e.g., exercise), aging, or disease state [40]. The raw score was used to asses fluid cognition and is the approach recommended by the developers for simple improvement or decline [40]. Assessment of individual cognitive constructs were also performed for processing speed, episodic memory, executive function, attention, and working memory. The NIH Toolbox assessment for fluid cognition has been validated among healthy adults ages 20–85 [41].

Serum Brain Derived Neurotrophic Factor (BDNF) Measurement: A 15 mL blood sample was collected at the completion of each trial (control, MIE, HIE, and yoga) in serum tubes (BD Biosciences, Franklin Lakes, NJ, USA) and centrifuged (968 *g*, 30 min, 4 °C). The serum portion of the whole blood was transferred to sterile microtubes and stored at −80 °C until analysis of serum BDNF. Concentration was measured using an enzyme-linked immunosorbent assay (ELISA) with a detection range of 15.6–1000 pg/mL (R&D Systems, Minneapolis, MN, USA). A 50-fold sample dilution was used and all manufacturer directions were followed. 

Whole Blood Lactate Measurement: A lancet puncture to the earlobe was used to collect a droplet of blood (~1 μL) for measurement of whole blood lactate. The droplet of blood was inserted into a handheld analyzer device (Nova Biomedical, Waltham, MA, USA). Samples were measured in duplicate and are reported in mmol/L (Hart et al. 2012). Prior to each trial, the handheld device was calibrated following manufacturer instructions. 

Statistical Analysis: Sample size was determined based on *a priori* calculation with power set to 0.80 and alpha level of 0.05 (G*power, Dusseldorf, Germany). The criterion for selected studies was acute exercise interventions and changes in Hbdiff using functional near infrared spectroscopy technology [2,4,20]. The estimated effect size was 1.10, which estimated a minimum of six subjects to detect a difference using the analyses selected. All results are expressed as means ± SD and checked for homogeneity of variance and normality. The Hbdiff (μmol) was analyzed from the RPFC and LPFC. Data from leftmost side of the frontal region (fNIRS channels 1–4) and rightmost region (fNIRS channels 5–8) were combined to represent LPFC and RPFC, respectively [2]. One-way ANOVA with repeated measures was used to analyze Hbdiff changes in the LPFC and RPFC between trials conditions (4 levels: control, MIE, HIE, YE) and used to evaluate the effect of exercise on regional PFC Hbdiff (2 levels: LPFC and RPFC). Post hoc Tukey analysis was performed when main effects were detected. Repeated measures one-way ANOVA was also used to compare between trial (control, MIE, HIE, YE) differences in cognitive constructs from the NIH Toolbox. Follow-up post hoc analyses were performed upon main effect finding. Pearson correlation analyses were performed to evaluate relationships between various exercise intensity markers (e.g., %HRmax, exercise lactate) and cognitive results. Pearson correlation was used to evaluate relationships between cognitive outcomes and PFC (both LPFC and RPFC) activation via fNIRS. Data were analyzed using IBM SPSS Statistics (version 25.0, Chicago, IL, USA).

## 3. Results

Subject and Trial Characteristics: Subject height, body mass, body composition, and maximal oxygen consumption are shown in Table 1. Average age for the participants was 35 ± 5 years with an average BMI in the “normal” classification (23.91) [42]. Functional capacity was used as an indicator of cardiorespiratory fitness. Male subjects had an average VO_2_max of 41.60 mL/kg/min, categorizing them into the “fair” classification for age and gender [42]. The average VO_2_max among female participants was 37.88 mL/kg/min, indicating “good” classification for fitness based on age and gender [42]. Based on fitness and BMI, we determined subjects in the current study to be healthy adults. 

Subjects completed all study trials, including control, HIE, MIE, and YE in randomized order. Exercise intensity indicators (VO_2_, heart rate, %HRmax, and lactate) were higher (*p* < 0.05) in the HIE compared with MIE and YE. MIE was higher (*p* < 0.05) in each intensity variable compared with YE. Table 2 shows the exercise intensity indicators for each trial.

**Figure 2:**
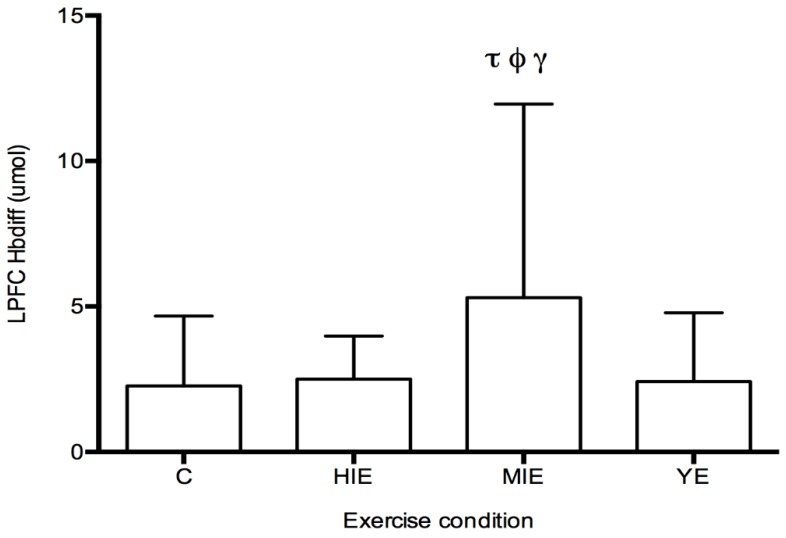
Left prefrontal cortex hemoglobin difference (Hbdiff) levels after control and exercise conditions. An increase in oxygenation as reported by Hbdiff was higher after MIE compared with all other conditions. τ significantly higher than control, *p* < 0.05. ф significantly higher than high intensity exercise, *p* < 0.05. γ significantly higher than yoga exercise, *p* < 0.05. LPFC—left prefrontal cortex, Hbdiff—hemoglobin difference, C-control, HIE—high intensity exercise, MIE—moderate intensity exercise, YE—yoga exercise. Data are mean ± SD, *N* = 8.

Prefrontal Cortex Hbdiff during Cognitive Testing: The fNIRS measurement of Hbdiff took place while participants performed the NIH Toolbox fluid cognition test, which was completed either during the control trial, or 20 min after HIE, MIE, and YE trials. Regions of interest were the LPFC and RPFC. No differences in LPFC and RPFC were observed for individual cognitive tests. A significant main effect was detected for Hbdiff in the LPFC (F_(3,87)_ = 4.63, *p* < 0.05, η^2^ = 0.131, Figure 2) during the entire cognitive assessment. Post hoc testing revealed significantly higher Hbdiff during the cognition test after MIE compared with control (5.30 ± 6.65 vs. 2.26 ± 2.40 μM, *p* < 0.05), high intensity exercise (5.30 ± 6.65 vs. 2.50 ± 1.48 μM, *p* < 0.05), and yoga exercise (5.30 ± 6.65 vs. 2.41 ± 2.36 μM, *p* < 0.05). No main effect was detected for the Hbdiff in the RPFC using fNIRS. No main effect was detected for regional differences (LPFC vs. RPFC). In all exercise conditions (HIE, MIE, and YE), Hbdiff in the LPFC and RPFC was statistically higher during the cognitive testing compared to the pre-exercise measurement (data not shown).

Post-Exercise Cognitive Performance: No differences were detected between trials (control, HIE, MIE, and YE) for any of the cognitive constructs measured by the NIH Toolbox test (Table 3). The assessment included: attention, processing speed, episodic memory, working memory, executive function, and overall fluid cognition.

**Figure 3:**
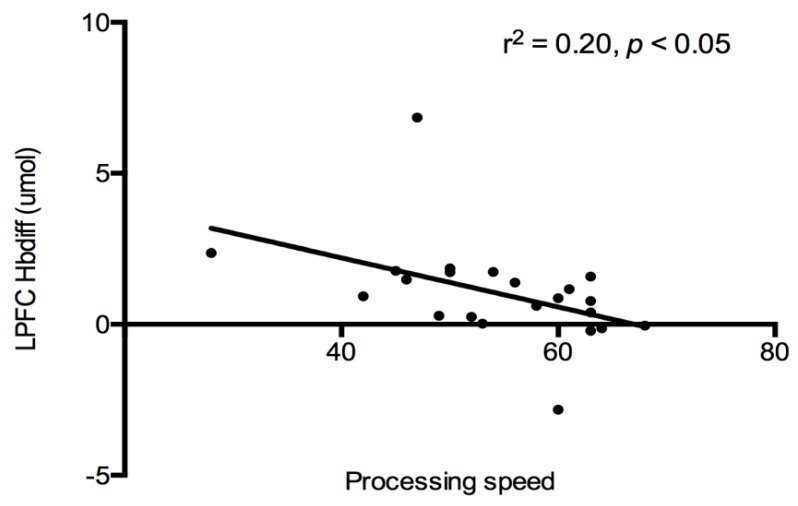
Relationship between LPFC Hbdiff and processing speed. A significant negative relationship was detected. LPFC—left prefrontal cortex, HBdiff—hemoglobin difference. *N* = 24.

Cognition and PFC Hbdiff: A correlational analysis was used to evaluate the relationship between cognitive constructs (see Table 2), and PFC (both LPFC and RPFC) oxygenation for each trial. No associations were detected between PFC regions and cognitive constructs after each exercise trial. All exercise data were combined together, and a significant negative relationship was detected between LPFC Hbdiff and processing speed (r^2^ = −0.20, *p* < 0.05, Figure 3). Indicating lower LPFC oxygenation was associated with higher processing speed scores after exercise. This relationship was lost when the control trial was added to the analysis. 

**Figure 4:**
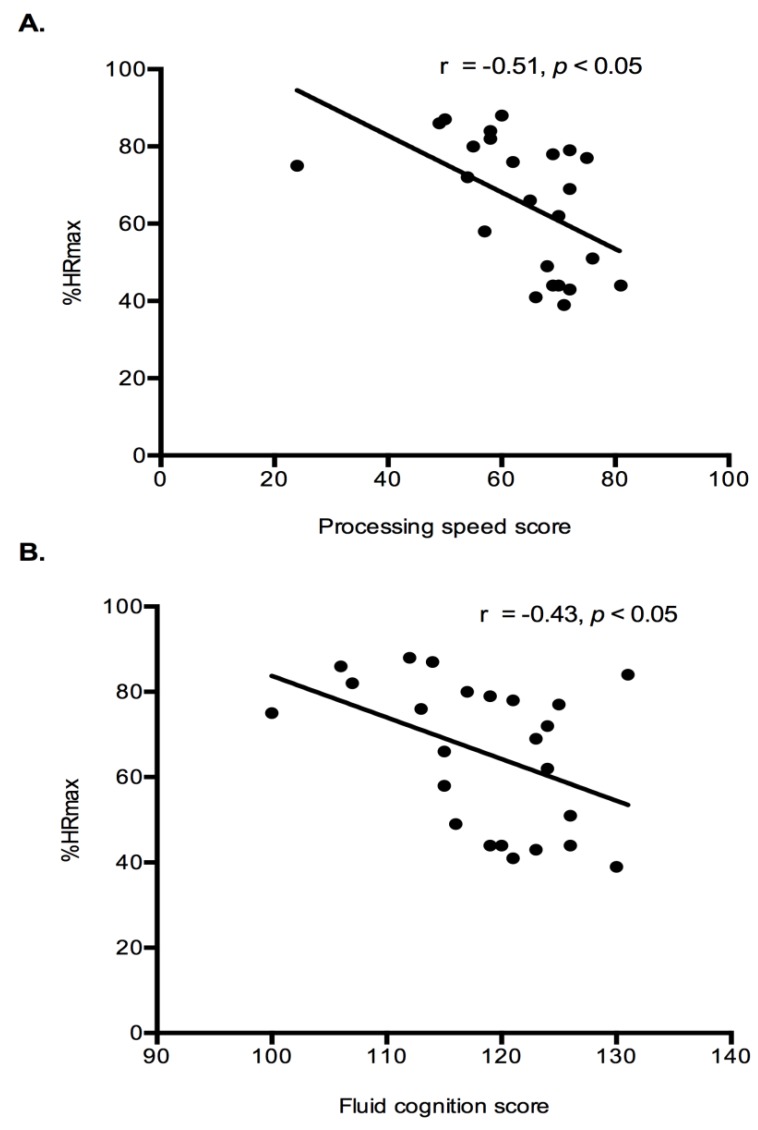
Relationship between exercise intensity and processing speed and fluid cognition. A significant negative relationship was detected between %HRmax and processing speed (**A**) and a similar relationship was observed for %HRmax and fluid cognition (**B**). %HRmax—percentage of maximal heart rate. *N* = 24.

Exercise Intensity Markers and Cognition: Correlation analysis were performed to determine if relationships existed between exercise intensity markers (%HRmax and lactate) and cognition results (fluid cognition and processing speed). Both fluid cognition (r = −0.43, *p* < 0.05) and processing speed (r = −0.46, *p* < 0.05) had a significant negative relationship with %HRmax (Figure 4). Indicating that higher intensity exercise was associated with lower cognitive performance. No relationships existed between the lactate response during exercise and any cognitive constructs.

**Figure 5:**
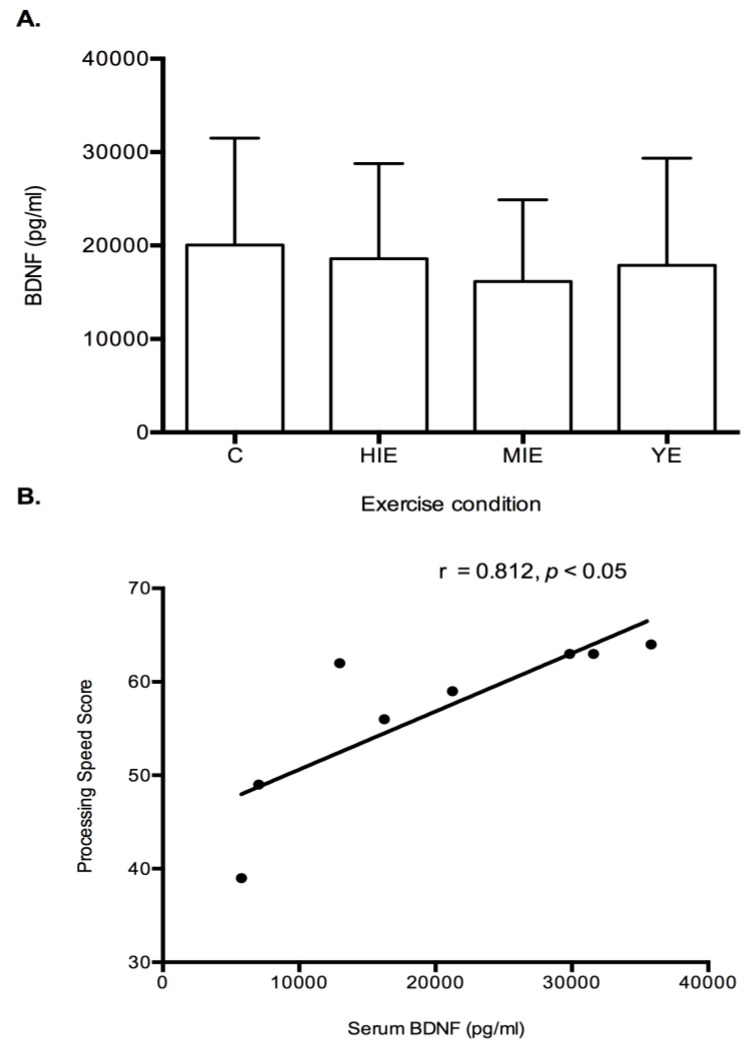
Serum BDNF in control and after exercise. No changes in serum BDNF were detected between conditions (**A**). A significant relationship was observed between control expression of serum BDNF and processing speed (**B**). BDNF—brain derived neurotrophic factor. Data are mean ± SD, *N* = 8.

Serum Brain Derived Neurotrophic Factor: No differences were detected between trials for serum BDNF levels (Figure 5A). Correlational analyses were performed to analyze the relationship between serum BDNF and cognitive constructs. A significant positive relationship was detected for baseline serum BDNF and processing speed (r = 0.812, *p* < 0.05, Figure 5B).

## 4. Discussion

The key finding in the current study is that PFC oxygenation and cognitive function are influenced by the intensity of exercise performed prior to a cognitive test battery. Specifically, LPFC oxygenation during cognitive testing was higher after completion of moderate intensity aerobic exercise compared with sedentary control, yoga, and high intensity efforts. The increase in cortical oxygenation did not lead to an increase in cognitive performance. However, a negative relationship was detected between PFC oxygenation and processing speed when all exercise conditions were combined (HIE, MIE, and yoga), and may indicate a small improvement in neural efficiency. More specifically, less neural input was required for a given processing speed result. Another interpretation is that for a given output, less input was needed. A negative relationship was also detected between cognitive constructs (fluid cognition and processing speed scores) and exercise intensity (%HRmax) showing that higher intensity exercise correlated with reduced mental performance. 

Monitoring the prefrontal cortex using functional near-infrared spectroscopy (fNIRS) imaging technology has been used in previous exercise and cognition studies [15]. Specific sites within the PFC, including the DLPFC, medial PFC, and ventral lateral PFC are thought to control cognitive processes such as working memory, processing speed, and overall executive function [43,44]. Higher activation in PFC regions occurs during cognitive challenging tasks and possibly indicates greater mental effort [6]. An increase in oxygenation detected by fNIRS technology is thought to demonstrate a rise in neural metabolic activation [45]. In the current study, post-exercise cortical oxygenation of the LPFC during cognitive testing increased only after completion of a 45-min bout of moderate intensity exercise. Similar to our results, other research groups have reported elevated left DLPFC and medial PFC oxygenation during cognitive testing after aerobic exercise ranging from 30%–60% of HRmax or peak workload for a duration of 10–15 min [4,20,46,47]. Of note, the yoga exercise herein was performed at 44% of HRmax, but did not lead to an increase in PFC oxygenation. Conversely, previous studies have shown that a single bout or short duration (e.g., 10 min) high intensity exercise has also been shown to promote cortical activation in the left DLPFC [2,8]. It is difficult to explain why various intensities or types of exercise may cause differential changes in post-exercise cortical activity. Short duration aerobic exercise intensity up to 80% of maximal workload appears to increase PFC oxygenation during exercise and thus cause post-exercise neural stimulation [48,49]. However, when exercise is below 50% workload, PFC oxygenation is not influenced by exercise. This may indicate a possible intensity threshold, and may be the reason that yoga exercise did not elicit a PFC response. When exercise approaches high intensity, or near maximal effort, hypofrontal oxygenation occurs as resources are shifted toward brain regions of motor and sensory function [50,51]. However, the hypofrontality response may also lead to an increase in post-exercise neural stimulation as a possible rebound effect may occur in brain regions [8,17]. One explanation for the differing responses in the current study may be a result of the duration of the exercise bouts. The majority of exercise studies examining PFC oxygenation have implemented shorter duration protocols. From this perspective, the high intensity bout may have been too long and the yoga session too short. However, we did not monitor PFC oxygenation during exercise in the current study to make this determination.

The detected increased oxygenation in the LPFC region in the current study did not align with an increase in post-exercise cognitive performance among the healthy middle-aged adult participants. We did identify a negative association between LPFC oxygenation and the processing speed construct when all exercise conditions were combined. This relationship may indicate neural efficiency changes produced by exercise regardless of type or intensity. Curtin et al., recently reported lower task-evoked activation in the left DLPFC with simultaneous improvements in processing speed in response to cognitive training [13]. This demonstrates an improvement in neural efficiency as the brain workload is reduced for a given output, and may explain how learning occurs in response to mental training [6]. In addition, this response may help to further the understanding of how an acute bout of exercise leads to a transient adjustment brain function. The dissociation reported herein might also signify that PFC oxygenation is not responsible for cognitive changes linked to exercise. However, other research groups have observed an increase in PFC oxygenation, which aligned with improvements in processing speed and executive function [46,52]. This occurrence indicates that exercise promotes higher neural activation and further leads to greater mental function. Conversely, an increase in post-exercise PFC oxygenation has also been reported along with no change in reaction time [2]. This scenario reveals worsening neural efficiency as more neural activation is required for the same cognitive output. Unfortunately, previous authors did not examine the relationship between PFC oxygenation and cognitive outcomes. 

Several possible reasons may explain the discrepancies between the previous studies and the current findings. First, a global assessment of fluid cognition was administered in our study, and included executive function, working memory, episodic memory, inhibitory control, attention, processing speed, and fluid cognition. Other research groups have used a single cognitive measure such as inhibitory control, working memory, or executive function [2,8,20]. The completion of the entire battery at once may have influenced individual testing results, along with PFC oxygenation changes. Further, the results of various cognitive tests may have also been influenced by a ceiling effect, or maximization of the test, and may indicate that additional brain activation was not required [53]. On the other hand, the NIH Toolbox cognitive battery was developed with the intention of briefly and accurately measuring fluid cognitive abilities [39]. This tool may provide a more thorough evaluation of the effects of exercise on cognitive performance. Second, the negative relationship was only detected between processing speed and LPFC oxygenation, and has not been previously assessed in response to exercise. This specific association may provide a strong indicator of neural efficiency changes as a result of an intervention [13]. Lastly, the post-exercise delay before cognitive function measurements was 20 min in the current study. If PFC oxygenation mediates the cognitive effects of exercise, then the stimulatory impact of exercise may have been lost during this period. The 20 min time frame was selected based on meta-analytical results of acute exercise on cognition [1]. 

Another factor related to acute exercise-induced changes in brain activation is lactate release from the contracting muscle. Lactate may provide an alternative nutrient to glucose for brain astrocytes, and serve as a precursor for glutamate release [54,55]. Glutamate is the main excitatory neurotransmitter in the brain, and its regulation has been linked to learning and cognitive impairment in conditions of psychosis and aging [56]. Lactate has also been linked to the induction of neural plasticity-genes, and further maintenance of long-term potentiation, which is a measure of memory and learning in animal studies [57,58]. Animals who were injected with lactate demonstrated similar upregulation of brain plasticity markers as animals exercised at lactate threshold [59]. These reasons might help to explain how higher intensity exercise facilitates cognitive improvement, but the lactate response in the current study was not associated with cognitive performance. 

Another proposed link between higher intensity exercise and cognition is through BDNF induction. Secretion of cathepsin B and Irisin from the contracting muscle have been shown to stimulate BDNF production and improve memory after exercise [60,61]. In humans, serum BDNF levels have increased from high intensity exercise, and correlated with improved short-term memory [26]. In addition, lactate released during high intensity exercise may also lead to BDNF production in human astrocytes [62]. No changes were detected for serum BDNF after an acute bout of exercise in the current study. A relationship between control (resting) levels of BDNF and processing speed was however detected (Figure 5B). This possibly indicates a higher resting value of BDNF is linked to cognitive functioning, but acute exercise in the current study did not enhance BDNF levels. Therefore, chronic exercise that elicits lactate release may promote BDNF expression, and further lead to long term cognitive changes. Monitoring of serum BDNF may be more beneficial in response to exercise training studies. 

While previous researchers have reported improvements in cognitive function after high intensity exercise, we observed no change. However, a negative association between higher intensity exercise (reported as %HRmax) and post-exercise processing speed and fluid cognition was detected [2,8]. Results regarding the influence of exercise intensity on post-exercise cognitive function are mixed. Both improvements and no changes in post-exercise reaction time in response to increased exercise intensity have been posted [63,64]. For example, Wohlend et al., recently posted a linear improvement in reaction time with increased exercise intensity from 63% HRmax up to 91% among a group with high aerobic fitness (54 mL·kg·min^−1^) [63]. Similarly, reaction time improved after strenuous exercise among endurance trained athletes [65]. Contrarily, no difference in reaction time was found after 20 min of exercise at either 50% or 75% VO_2_max among a group of elderly women. One explanation for differing results is the lack of standardization of energy expenditure in exercise and cognition studies [63]. When controlling for energy expenditure, more intense, and shorter exercise appears to improve reaction time [63]. In the current study, energy expenditure was not controlled in effort to compare various types of exercise (HIE, MIE, yoga), so it is likely that the high intensity group expended more energy. High intensity exercise causes physical fatigue, which is related to declining cognitive function, and may explain why the negative association was detected [66,67]. Another possible explanation is related to the participant’s cardiovascular fitness and experience with performing high intensity exercise. Those with higher fitness and more exercise experience should tolerate and recover better from intense exercise compared those with lower physical fitness and less experience. 

The benefits of exercise among those suffering from psychological or behavioral disorders, along with traumatic brain injury has expanded, but is incomplete. Various forms of exercise have been shown to alleviate symptoms of depression and anxiety [68]. Patients suffering from schizophrenia spectrum disorders have not only demonstrated improvement in clinical outcomes, but also experienced improved brain plasticity after aerobic exercise [69]. Transient neural changes in response to acute exercise may promote an exercise priming effect for cognitive or motor related tasks [70,71,72]. The results of current study demonstrate that exercise (regardless of type) improves neural efficiency. When extended into clinical populations, the improvements in mental processing as a result of exercise will allow a patient to more fully engage in, and benefit from a cognitive or motor task. This approach has been used among stroke patients in which a brief 20–30-min bout of aerobic exercise improved retention of motor skills learned from physical therapy [71]. Exercise interventions have also been implemented prior to learning objectives among children and adolescents with positive results reported [73]. The application of the exercise priming affect may extend into other conditions and treatments. For example, brief exercise prior to a cognitive behavioral therapy session may improve the retention of skills taught, and increase the success of such treatments. These considerations and applications for exercise should be considered in future studies.

Several limitations in the current study must be considered when interpreting the reported results. First, the low sample size and target population (healthy adults) may undermine the external validity of the study. Secondly, other cortical sites were not measured during exercise, which may have provided additional insight into the post-exercise responses. 

In conclusion, moderate intensity exercise increased cortical oxygenation during post-exercise cognitive testing, but this response was not linked to improvements in cognitive performance. However, a negative relationship was detected for LPFC oxygenation and processing speed when all exercise conditions were combined. This represents a possible acute adjustment in neural processing, and provides additional insight into how acute exercise effects brain function. Lastly, higher intensity exercise was associated with lower processing speed and fluid cognition scores. This indicates that fatiguing exercise may be detrimental on subsequent cognitive processes.

## Figures and Tables

**Table 1:** Subject characteristics. Mean ± SD.

Characteristic	*N* = 8 (4 Male, 4 Female)
Age	35 ± 5
Height (cm)	168.80 ± 10.22
Weight (kg)	68.50 ± 10.71
Body mass index (kg·m^2^)	23.91 ± 1.69
Body fat (%)	21.51 ± 5.05
VO_2_max (mL·kg·min^−1^)	39.74 ± 7.24

**Table 2:** Exercise intensity measurements. Mean ± SD for each trial.

Measurement	High Intensity	Moderate Intensity	Yoga
Duration (min)	45	45	45
RPE	16 ± 2	12 ± 1	9 ± 1
Heart rate (bpm)	150 ± 8 *^#^	129 ± 12 ^#^	81 ± 6
%HRmax	81 ± 5% *^#^	70 ± 8% ^#^	44 ± 3%
Oxygen consumption (VO_2_, mL·kg·min^−1^)	27.70 ± 5.2 *^#^	21.37 ± 4.19 ^#^	4.08 ± 78
Lactate (mmol/L)	11.36 ± 2.73 *^#^	4.05 ± 1.46 ^#^	1.58 ± 62

HR—heart rate. * statistically different from moderate intensity, *p* < 0.05. ^#^ statistically different from yoga, *p* < 0.05.

**Table 3:** Results from the NIH Toolbox fluid cognition test (corrected T-score).

	Control	Moderate Intensity	High Intensity	Yoga
Cognitive Test (Construct)				
Flanker (attention)	45 ± 6	44 ± 4	44 ± 3	46 ± 7
Card sort (executive function)	49 ± 12	52 ± 10	54 ± 7	53 ± 12
Picture sequence (episodic memory)	69 ± 17	60 ± 10	69 ± 13	69 ± 12
Work memory (working memory)	53 ± 9	57 ± 8	54 ± 10	55 ± 6
Pattern comparison (processing speed)	64 ± 16	57 ± 17	58 ± 7	70 ± 3
Fluid cognition	120 ± 9	115 ± 8	118 ± 7	122 ± 4

Mean (SD), *N* = 8.
